# Single-Molecule Detection of the Encounter and Productive Electron Transfer Complexes of a Photosynthetic Reaction Center

**DOI:** 10.1021/jacs.4c03913

**Published:** 2024-07-11

**Authors:** Cvetelin Vasilev, Jon Nguyen, Adam G. M. Bowie, Guy E. Mayneord, Elizabeth C. Martin, Andrew Hitchcock, Taras V. Pogorelov, Abhishek Singharoy, C. Neil Hunter, Matthew P. Johnson

**Affiliations:** † Plants, Photosynthesis and Soil, School of Biosciences, University of Sheffield, Firth Court, Western Bank, Sheffield S10 2TN, U.K.; ‡ School of Molecular Sciences, Arizona State University, Tempe, Arizona 85281, United States; § Department of Chemistry, Center for Biophysics and Quantitative Biology, Beckman Institute for Advanced Science and Technology, National Center for Supercomputing Applications, School of Chemical Sciences, University of Illinois Urbana−Champaign, Urbana, Illinois 61801, United States

## Abstract

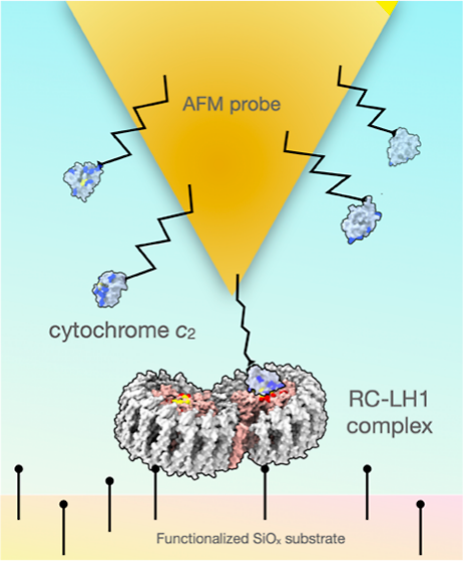

Small, diffusible redox proteins play an essential role in electron transfer (ET) in respiration and photosynthesis, sustaining life on Earth by shuttling electrons between membrane-bound complexes via finely tuned and reversible interactions. Ensemble kinetic studies show transient ET complexes form in two distinct stages: an “encounter” complex largely mediated by electrostatic interactions, which subsequently, through subtle reorganization of the binding interface, forms a “productive” ET complex stabilized by additional hydrophobic interactions around the redox-active cofactors. Here, using single-molecule force spectroscopy (SMFS) we dissected the transient ET complexes formed between the photosynthetic reaction center-light harvesting complex 1 (RC-LH1) of *Rhodobacter sphaeroides* and its native electron donor cytochrome *c*_2_ (cyt *c*_2_). Importantly, SMFS resolves the distribution of interaction forces into low (∼150 pN) and high (∼330 pN) components, with the former more susceptible to salt concentration and to alteration of key charged residues on the RC. Thus, the low force component is suggested to reflect the contribution of electrostatic interactions in forming the initial encounter complex, whereas the high force component reflects the additional stabilization provided by hydrophobic interactions to the productive ET complex. Employing molecular dynamics simulations, we resolve five intermediate states that comprise the encounter, productive ET and leaving complexes, predicting a weak interaction between cyt *c*_2_ and the LH1 ring near the RC-L subunit that could lie along the exit path for oxidized cyt *c*_2_. The multimodal nature of the interactions of ET complexes captured here may have wider implications for ET in all domains of life.

## Introduction

Electron transfer (ET) reactions between small, mobile redox carrier proteins and their larger membrane integral redox partners underpin both respiration and photosynthesis, and therefore life on Earth.^1^ The ET chains found in mitochondria, chloroplasts and photosynthetic bacteria are located within specialized, invaginated membranes that confine these small redox proteins within a lumen that promotes rapid diffusion and millisecond, reversible interactions with multiple partners. One example includes cytochrome (cyt) *c*-mediated ET between the cyt *bc*_1_ and *aa*_3_ oxidase complexes in the cristae of mitochondria;^2−4^ in another instance, plastocyanin (*Pc*) sits within the lumen formed by stacked, flattened thylakoids found in cyanobacteria and chloroplasts of plants and algae, shuttling electrons from the cyt *b*_6_*f* complex to photosystem I (PSI).^5,6^

For each donor–acceptor pair there is a specific and rapid association between membrane-bound and extrinsic redox proteins followed by ET. Dissociation and diffusion of the mobile electron carrier to the next component then sustains directionality and high turnover of the ET chain. These ET components typically employ hemes, chlorophylls or iron–sulfur centers, and the gain or loss of an electron from these cofactors can be monitored by changes in absorption. Past kinetic, structural and modeling studies show that the productive ET complex forms in two distinct stages. In the first, long-range electrostatic interactions steer the approach of the soluble redox protein toward its membrane integral partner, leading to formation of a “loose” encounter complex. In the second, short-range hydrophobic interactions then form through reorganization of the binding interface and exclusion of the intervening water molecules, promoting the close association of the reduced and oxidized cofactors and thus efficient and productive ET.^7−13^

Studying the formation of the encounter and ET complexes presents some challenges, given its transience and the relatively weak interactions that sustain it. Nuclear magnetic resonance (NMR) spectroscopy of the cyt *c*–cyt *b*_5_ ET complex has shown an extensive interaction interface between these proteins with a dynamic ensemble of structures.^14^ More recently, analysis of cryogenic electron microscopy (cryo-EM) data sets of the mitochondrial III_2_IV supercomplex revealed the density of interacting cyt *c* was “blurred”, indicating a range of binding conformations that could be resolved using three-dimensional variability analysis.^15,16^

Single-molecule force spectroscopy (SMFS) utilizing an atomic force microscope (AFM) provides a different approach for studying the transient interactions that govern biological ET complexes. This is achieved using a scanning probe functionalized with one of the ET proteins, which is lowered toward a surface-tethered partner. ET can occur when the tip and surface-attached redox proteins are brought into contact, and the forces that mediate the docking and/or undocking of the probe-attached ET partner can be quantified. An implicit feature of this method is that ET at a membrane surface is governed by a series of single molecule interactions and probabilities, rather than being driven by ensemble effects such as concentrations. The SMFS approach has been applied to ET partners in the purple phototrophic bacterium *Rhodobacter* (*Rba*.) *sphaeroides*, which provides a useful model for light-triggered ET at a membrane interface.^17,18^ This bacterium contains hundreds of spherical invaginations (chromatophores), ∼50 nm in diameter.^19,20^ Confinement of ∼12 cyt *c*_2_ molecules within the lumen of the vesicle promotes fast ET between reaction center light-harvesting complex 1 (RC-LH1) and cyt *bc*_1_ complexes at the membrane surface.^21^ RC-LH1 complexes receive excitation energy from nearby LH2 complexes, and a special pair of BChls in the RC undergoes a charge separation followed by a cascade of ET reactions that culminate in the reduction of a quinone acceptor (Swainsbury et al., 2023). The electron “hole” in the RC is filled when a reduced cyt *c*_2_ docks at the RC surface.^22,23^ After donating its electron, cyt *c*_2_ returns to the cyt *bc*_1_ complex to pick up another electron. SMFS has also been extended to the membrane localization and docking of *Pc* onto the cyt *b*_6_*f* complex,^24,25^ and to the binding of *Pc* to PSI.^26^ These studies show that opposing redox states (reduced/oxidized) of the cognate partners strongly influence the probability that they will form a complex, suggesting that ET complex interactions are redox gated as well as being steered by electrostatic interactions, consistent with molecular dynamics simulations.^13,27^ Such redox gating plays a role in minimizing unproductive encounters within the ET chain.

In order to study the initial and productive ET complexes in more depth we used the RC-LH1/cyt *c*_2_ ET complex as a model system. We made use of several RC-LH1 mutants with modified cyt *c*_2_ binding sites, which have been described and characterized previously by ensemble methods.^28^ These mutants introduce or reverse charges at various cyt *c*_2_ interaction sites on the lumenal face of the RC L- and M-subunits, with M184 and M188 closer to the site ET and L264 further away. This SMFS analysis is aided by the availability of structures of the monomeric and dimeric RC-LH1 complexes,^29,30^ and investigations of encounter and full ET complexes are augmented by steered molecular dynamics (SMD) and Brownian dynamics (BD) simulations.^22^ Together, these approaches quantify the intermolecular forces that stabilize the encounter and productive ET complexes formed between reduced cyt *c*_2_ and the photo-oxidized RC.

## Results

### SMFS Characterization of RC-LH1/Cyt *c*_2_ Interactions

**Figure 1:**
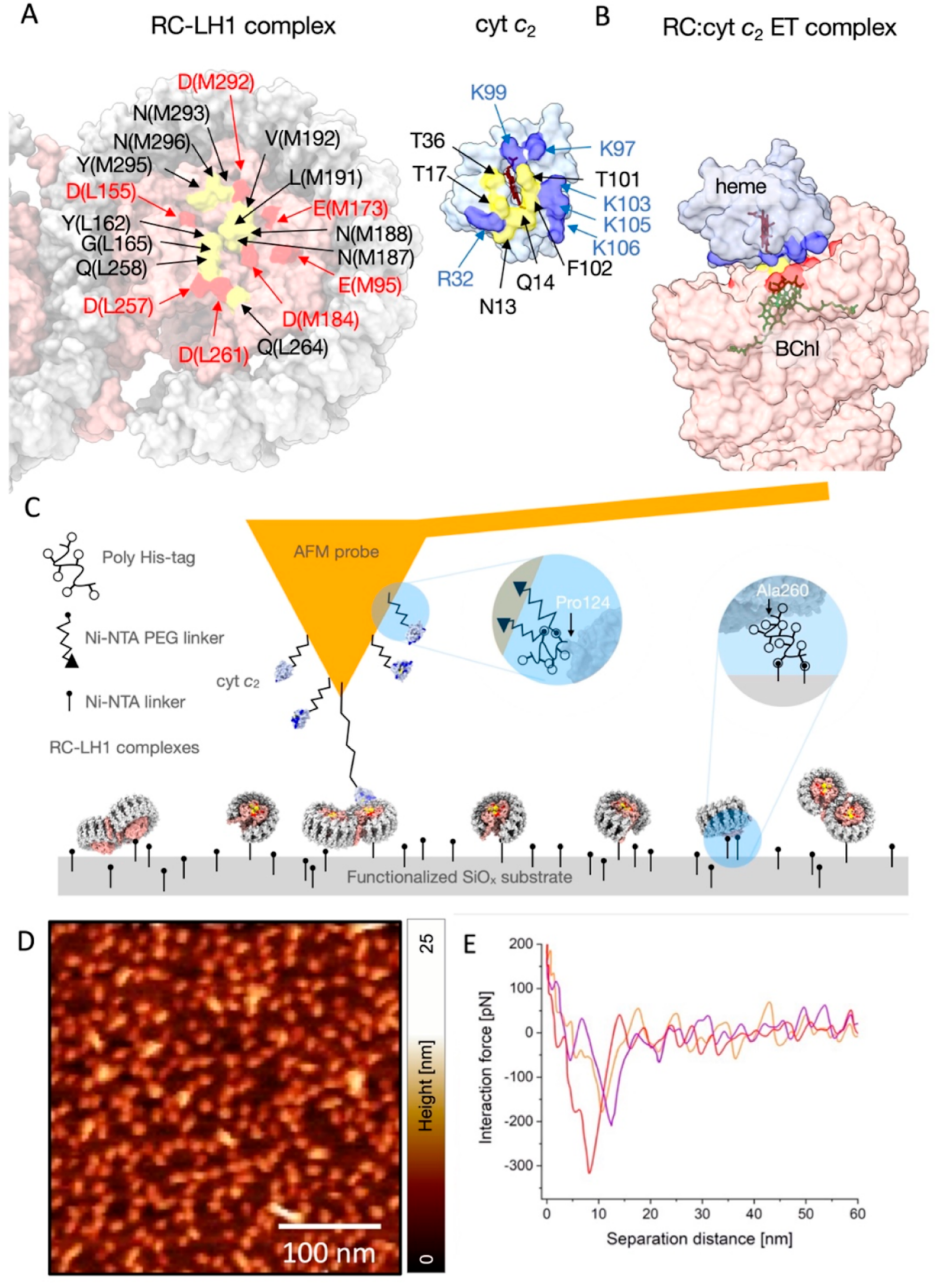
Affinity mapping of surface-attached wild-type (WT) RC-LH1 complexes using a cyt *c*_2-_modified AFM probe. (A) The lumenal surface of the RC-LH1 complex of *Rba. sphaeroides*, showing acidic residues (red) and uncharged residues (yellow) involved in the binding interface with cyt *c*_2_ (pale blue, with basic residues in blue). The RC is in pink and the surrounding LH1 complex is in gray. The binding interfaces for RC-LH1 and cyt *c*_2_ are displayed in an open book form (PDB: RC-LH1 dimer–7PIL; RC-cyt *c*_2_–1L9J). (B) The ET complex between RC-LH1 and cyt *c*_2_ with color coding of residues as in panel A. The heme (red) and bacteriochlorophyll (BChl) (green) ET cofactors are also highlighted. (C) Schematic representation of cyt *c*_2_ molecules attached to the AFM probe and RC-LH1 complexes attached to the functionalized SiOx substrate. The blue circled insets show protein attachment points, each of which is distal to the cyt *c*_2_–RC binding interface. In the case of cyt *c*_2_ a His_6_-tag follows the C-terminal residue Pro124, and then a PEG linker is used to attach the His-tag to the AFM probe. For RC-LH1, the C-terminal Ala260 of the RC H-subunit is followed by a 14-residue linker including a thrombin cleavage sequence and then a His_10_-tag. (D) AFM topography image (in liquid buffer) of individual WT RC-LH1 complexes on the SiOx substrate. The surface density is ∼300–500 molecules per μm^2^. (E) Typical force–distance curves that exhibit the specific interaction between RC-LH1 complexes and cyt *c*_2_ with a separation distance ∼10 nm.

The X-ray crystallographic structure of the RC/cyt *c*_2_ ET complex shows a binding interface featuring a hydrophobic site of ET, surrounded by a ring of charged residues on both partners (Figure 1A,B).^7^ Complementary electrostatic interactions are formed between negatively charged acidic residues (shown in red) on the RC-L subunit [D(L155), D(L257) and D(L261)], and RC-M subunit [E(M95), D(M173), D(M184) and D(M292)], and positively charged residues (shown in blue) R32, K97, K99, K103, K105, and K106 on cyt *c*_2_. Hydrogen bonds exist between Q(L258), N(M187) and N(M188) on the RC, and the backbone of residues K99, T101 and K103 on the cyt *c*_2_.^31^ Short-range hydrophobic interactions occur between Y(L162), G(L165), N(M188), L(M191), V(M192), N(M293), N(M296) on the RC, and N13, G14, T17, T36, T101, F102, plus the heme cofactor of cyt *c*_2_,^7,32^ all shown in yellow. This interface also includes a π-cation interaction between the aromatic ring of Y(M295) on the RC with R32 on cyt *c*_2_.^11,31,33^

To create the conditions for transiently forming an ET complex, we attached RC-LH1, bearing a His_10_-tag on the C-terminus of the RC H-subunit, onto a silicon oxide substrate functionalized with Ni^2+^-NTA via silane monolayer chemistry (Figure 1C). Using the same chemistry, His_6_-tagged cyt *c*_2_ complexes (tag added after Pro124) were attached to an AFM probe via a ∼10 nm long (PEG_24_) polymer linker. This approach, employing polyhistidine tags in combination with a flexible linker, allows both partner proteins to move and orient freely, thus exposing their binding interfaces and enabling their interaction (Figure 1C).

PeakForce quantitative nanomechanical mapping (PF-QNM), a type of SMFS, was used for surface mapping and quantification of cytochrome *c*_2_–RC interactions. This technique maps surface topography, while identifying and quantifying binding events. The topograph in Figure 1D was obtained using a cyt *c*_2_—modified AFM probe to map the distribution of preoxidized, surface-attached RC-LH1 complexes, showing that these complexes are homogeneously dispersed on the substrate. Each force–distance curve reflects the characteristic rupture events occurring when the two bound proteins are separated by the upward motion of the AFM probe (Figure 1E). The interaction between the two ET partners leads to an increase in the force required to separate them. The interaction forces are manifested as a series of spikes in the force–distance curves that are offset by ∼10–20 nm from the surface (corresponding approximately to the length of the flexible PEG linker, and the height of the RC from the surface), thus allowing an unambiguous distinction between specific and nonspecific interactions (Figure 1E). PF-QNM measures these separation forces at loading rates up to 2 orders of magnitude higher than for dynamic force spectroscopy, and with short tip–sample contact times in the 70–240 μs range. The baseline noise in the individual curves in Figure 1E is a consequence of the high modulation frequency used, but the large number of curves acquired, and the number of data points per curve, enable robust calculations of cumulative interaction probabilities.

### Effects of Varying Salt Concentration on the Bimodal Distribution of the RC-LH1/Cyt *c*_2_ Interaction Forces

Initially, the sample was imaged in a buffer containing 10 mM HEPES pH 7.4 supplemented with 10 mM NaCl to assess the distribution of the surface-bound core complexes. After inspecting the sample surface and finding an area with uniform coverage of RC-LH1 complexes, a SMFS data set was acquired using a cyt *c*_2_-functionalized probe. The data set consisted of a topography image and corresponding adhesion map. Unless otherwise stated, before starting the measurements the RC-LH1 complexes were preoxidized by adding 0.8 mM potassium ferricyanide solution to the imaging buffer (followed by a sample rinse in the imaging buffer) and the cyt *c*_2_ proteins were prereduced using reducing buffer (imaging buffer supplemented with 0.5 mM sodium dithionite and 0.25 mM phenazine methosulfate), followed by rinsing the AFM probe in imaging buffer.

**Figure 2:**
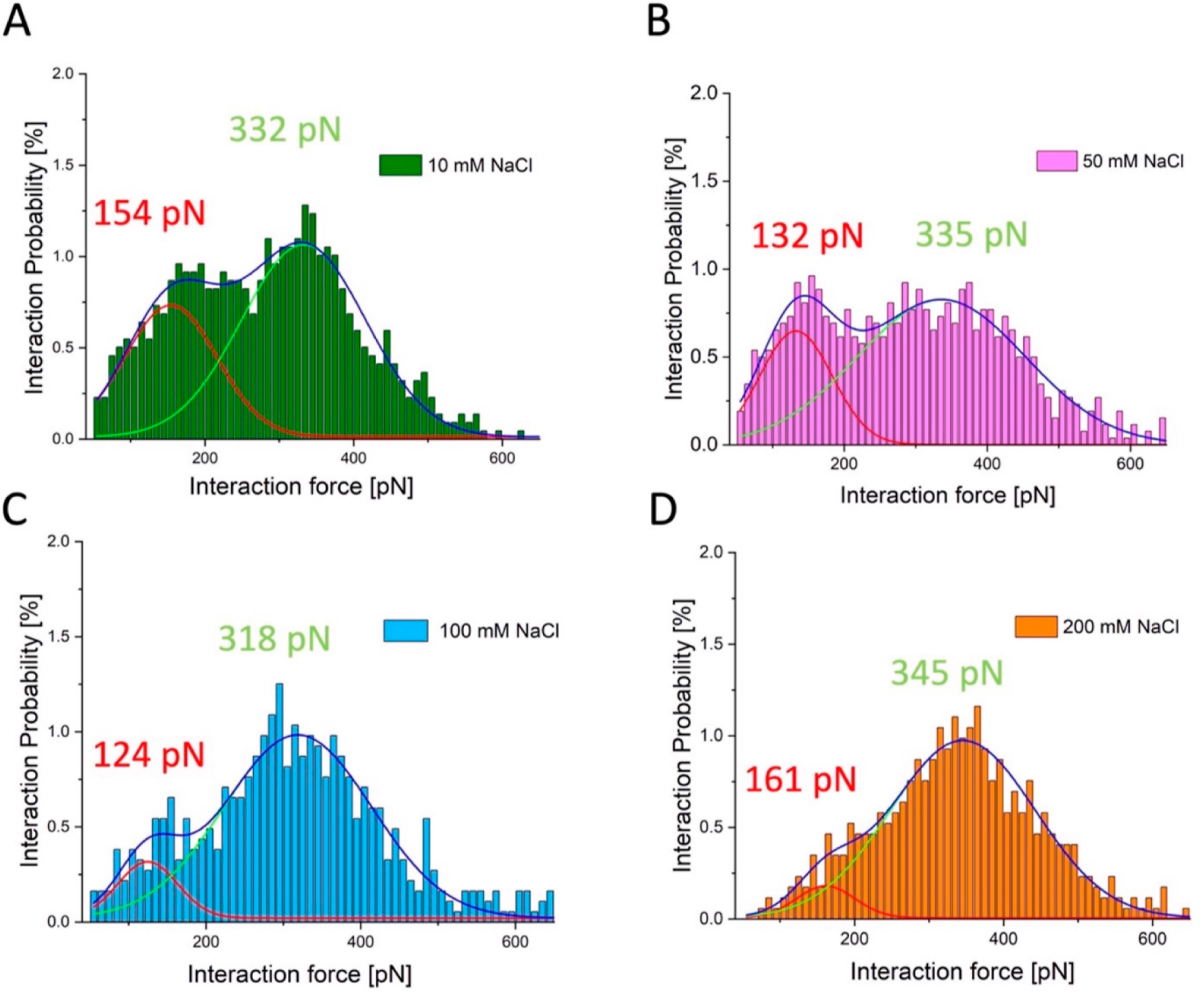
Distribution of the interaction force between oxidized WT RC-LH1 complexes and reduced cyt *c*_2_ versus the interaction probability. The histograms represent the distribution of the interaction forces measured by SMFS as oxidized WT RC-LH1 complexes and reduced cyt *c*_2_ are brought into contact and then separated. (A–D) The salt concentration in the imaging buffer was increased from 10 to 200 mM, as indicated next to each histogram. The solid curves represent the best fit for each of the histogram peaks, with the low force component in red, the high force component in green, and the cumulative fit in dark blue. The respective forces, in pN, are shown above each component.

Each data set was analyzed to select the force–distance curves displaying protein unbinding events, and to extract parameters such as interaction force, separation distance, and loading rate for each curve. The cumulative interaction probability was calculated as the ratio between the number of unbinding events (force–distance curves displaying rupture events) and the total number of RC-LH1 complexes imaged during the SMFS scan. The interaction force distribution was analyzed statistically to evaluate the most probable interaction force. Figure 2A shows the distribution of the interaction forces versus the interaction probability for oxidized RC-LH1 complexes imaged with a probe functionalized with reduced cyt *c*_2_ in an imaging buffer containing 10 mM NaCl. In this case, the interaction probability (i.e., the total area of the histogram) was approximately 34%. The histogram exhibits a clear bimodal distribution, which is unlikely to reflect multiple, simultaneous tip-sample interactions. Such events would occur with a lower probability than for single binding events, so a double force interaction could give rise to an apparent high force peak in the histogram but with a lower amplitude than for single interactions. Figure 2 shows that the high force component is often dominant in the overall force distribution; for 10 mM NaCl, there are two peaks at approximately 154 and 332 pN, in agreement with our earlier study that showed unbinding forces of 164 ± 19 and 305 ± 25 pN.^18^

**Figure 3:**
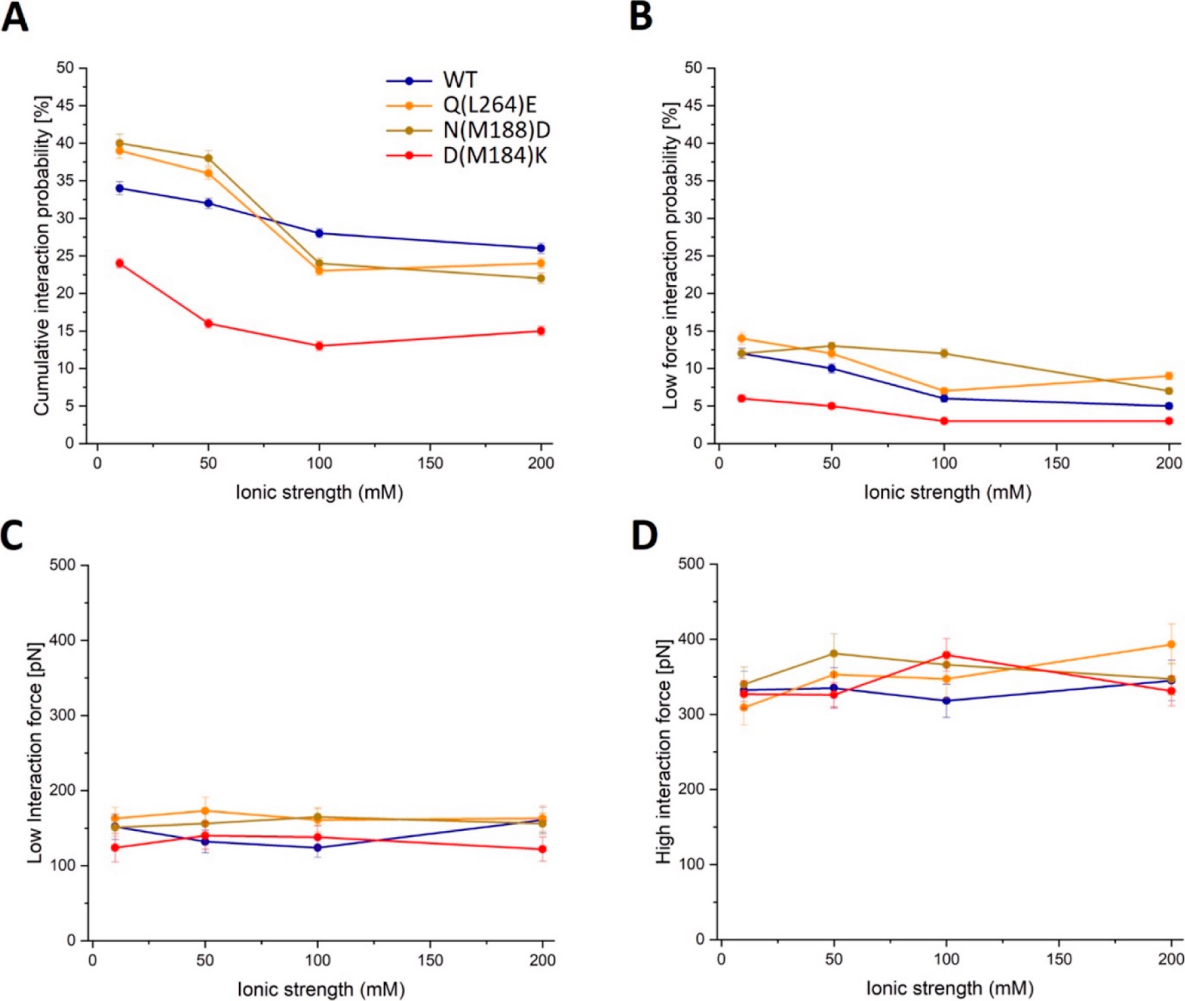
Force spectroscopy parameters in the WT versus D(M184)K, Q(L264)E and N(M188)D mutants of the RC-LH1 complex. The data were acquired upon the separation of complexes, initially brought into contact as oxidized RC-LH1 and reduced cyt *c*_2_, at different salt concentrations in the imaging buffer. (A) Cumulative interaction probability. (B) Probability of the low force interaction event. (C) Interaction force for the low-force peak. (D) Interaction force for the high-force peak. The WT and mutant RC-LH1 complexes are color coded in panel A.

Given the participation of acidic and basic residues in the RC/cyt *c*_2_ binding interface (Figure 1A), we investigated the rate of oxidation of reduced cyt *c*_2_ as a function of salt concentration by performing steady state RC-LH1 turnover assays with ubiquinone-2 as a terminal acceptor (Figure S1). The optimum salt concentration was approximately 100 mM, significantly higher than the 40 mM previously reported using non-native horse heart cyt *c*.^34^ The 100 mM optimum for the native RC-LH1/cyt *c*_*2*_ system studied here highlights the importance of electrostatic interactions in steering the formation of the initial encounter ET complex. This concentration is comparable with the 150 mM salinity used to model physiological conditions for the function of photosynthetic membranes in computational simulations of cyt *c*_2_–RC binding.^22^ To investigate the influence of salt concentration on the bimodal distribution of interaction force we conducted SMFS measurements in an imaging buffer with differing concentrations of NaCl. Multiple data sets were acquired for each salt concentration, and the peak positions and amplitudes of the high and low force components were determined by Gaussian fits and deconvolution of the histograms. The stepwise increase in the salt concentration from 10 to 200 mM NaCl (Figure 2B–D) does not alter the positions of the low and high force components, which remained relatively unchanged at 120–150 pN and 320–340 pN, respectively. The most striking effect revealed by the deconvolutions was the progressive and selective attenuation of the 120–150 pN low force component, and the gradual decrease in cumulative interaction probabilities, which were 34, 32, 28 and 26% at 10, 50, 100, and 200 mM NaCl respectively (Figure 2A–D). Thus, we assign this low force component to the encounter complex, which is established through long-range electrostatic interactions. In contrast, increasing salt concentration had less effect on the interaction probability of the high force component, suggesting that it arises from the short-range hydrophobic interactions that foster efficient ET within the productive complex. The differing responses of the low and high force components to ionic strength (see also Figure 3 below) negate the possibility that the high force component arises from a double RC/cyt *c*_2_ interaction, since varying ionic strengths would influence single and double interactions equally.

### Effects of Altering Charged Residues at the RC/Cyt *c*_2_ Binding Interface on the Bimodal Distribution of Interaction Forces

We explored this assignment further by mutating the cyt *c*_2_ binding interface of the RC-LH1 protein complex, through alteration of charged and polar residues on the lumenal face of the RC. These mutants, D(M184)K and N(M188)D and Q(L264)E (residue positions shown in Figure 1), have previously been reported to weaken or strengthen the interactions between the ET partners, and thus affect the rate of ET.^8,28^ These previous studies used flash-absorption spectroscopy to analyze RC-only preparations with no LH1 complex present; they showed that, compared to the WT core complex (*K*_D_ = 0.3 μM), the D(M184)K mutant is a weaker binder of cyt *c*_2_ (*K*_D_ = 250 μM) whereas the Q(L264)E (*K*_D_ = 0.01 μM) and N(M188)D (*K*_D_ = 0.06 μM) mutants have a higher affinity for cyt *c*_2_. Stronger binding enhanced the second order rate constant, whereas weaker binding decreased it.^28^ For each of these three cyt *c*_2_-binding interface mutants, examined here with the LH1 complex present, we measured the interaction forces and the interaction probabilities using SMFS at different salt concentrations.

As for the WT RC-LH1 complex (Figure 2), we also found a bimodal interaction force distribution for all three of the RC-LH1 mutants at all four salt concentrations analyzed (Figures S2–S4). However, relative to the WT, the cumulative interaction probability for the mutants responded differently to increasing salt concentration. For the higher affinity Q(L264)E and N(M188)D RC-LH1 mutants, the cumulative interaction probability was higher than the WT at 10 and 50 mM NaCl, and lower at 100 and 200 mM NaCl (Figure 3A), possibly because the increased salt concentration screens the effect of adding glutamic or aspartic acid side chains in these mutants. The lower cumulative interaction probability for the D(M184)K mutant at all NaCl concentrations (Figure 3A) is consistent with the greatly weakened binding of cyt *c*_2_, indicated by the approximately 800-fold increase in *K*_D_ measured by Tetreault et al.^28^

There is a consistent decrease in the interaction probability for the low force component for the D(M184)K mutant from 10 to 200 mM NaCl (Figure 3B). Therefore, the long-range electrostatic interactions associated with the encounter complex have been destabilized by reversing the charge at position M184. The opposite effect was seen for Q(L264)E and N(M188)D RC mutants, for which there was a small though consistent increase in the interaction probability for the low force component relative to the WT (Figure 3B). This result likely arises because Q(L264)E and N(M188)D increase the number of potential electrostatic interactions with the multiple Lys side chains on the heme-exposed face of the incoming cyt *c*_2_ (Figure 1A), promoting formation of the RC-LH1/cyt *c*_2_ ET complex (Figure 1B). As in the WT, in all mutants the low force component is attenuated more severely by increasing salt concentration relative to the high force component, consistent with the proposal that the latter includes additional contributions from short-range hydrophobic interactions (Figures S2–S4).

In contrast to the different interaction probabilities measured for the mutants relative to the WT (Figure 3A), the low and high force components each stayed within a narrow range with varying ionic strength (Figure 3C,D). The fact that alteration of surface charges in D(M184)K, Q(L264)E and N(M188)D did not affect the interaction forces substantially shows that while individual residues play a significant role in guiding the formation of the encounter complex they contribute only a small fraction of the overall binding energy, which is shared across the entire interface.

### Influence of RC and Cyt *c*_2_ Redox States on the Low and High Force Components

**Figure 4:**
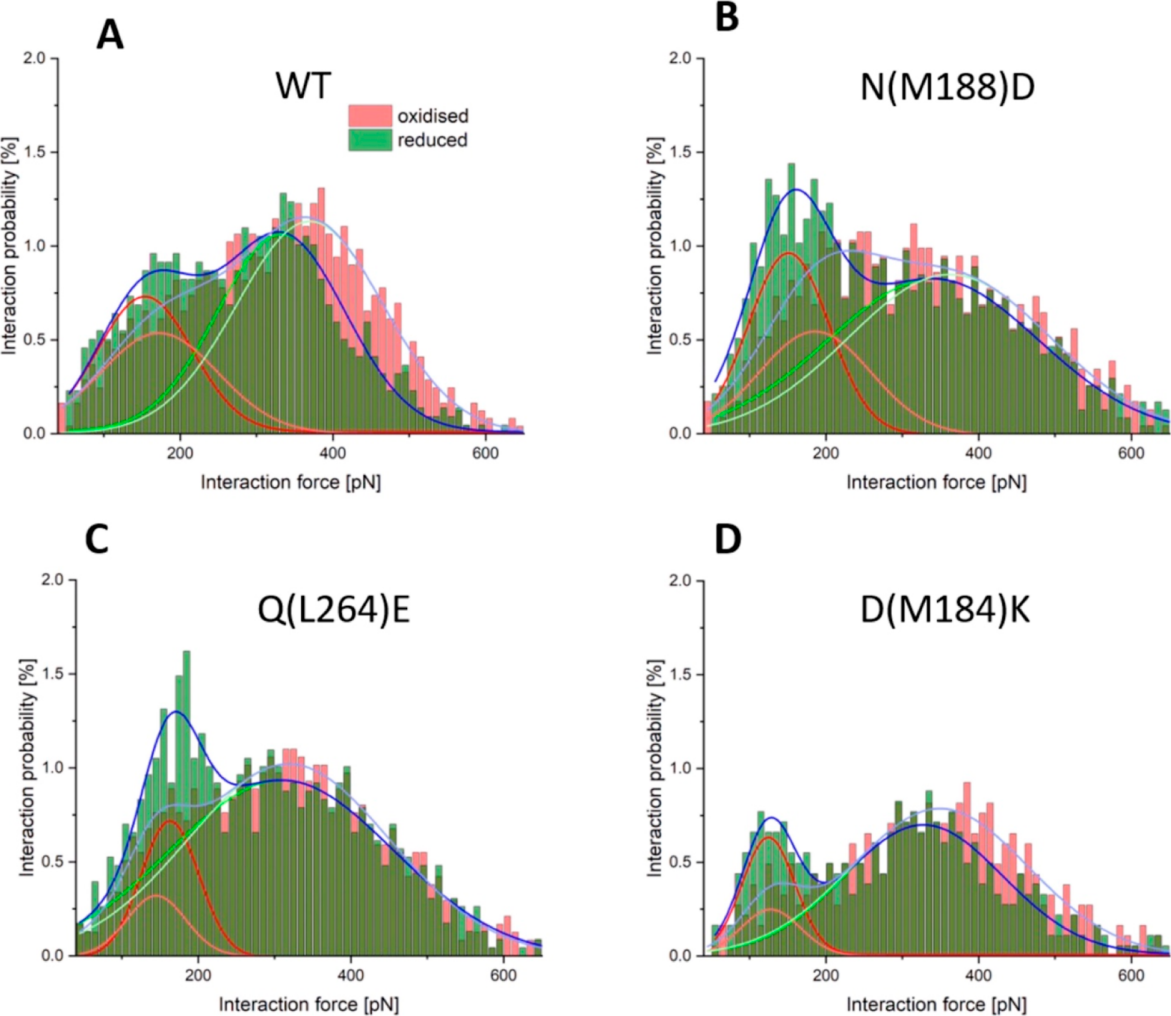
Force distributions for interactions between oxidized WT and mutant RC-LH1 complexes and oxidized or reduced cyt *c*_2_. (A–D) Histograms representing the distribution of the interaction forces measured at 10 mM NaCl using either reduced (green bars) or oxidized (red bars) cyt *c*_2_ as a partner for oxidized WT (A), N(M188)D (B), Q(L264)E (C) and D(M184)K (D) RC-LH1 complexes. The darker areas in each panel show the areas where the histograms for reduced cyt *c*_2_ and oxidized cyt *c*_2_ overlap. The solid curves in represent the best fit for each of the histogram peaks, with the low force component in red (reduced cyt *c*_2_) and orange (oxidized cyt *c*_2_), the high force component in green (reduced cyt *c*_2_) and olive (oxidized cyt *c*_2_), and the cumulative fit in dark blue (reduced cyt *c*_2_) and light blue (oxidized cyt *c*_2_).

Previously, it was shown that the redox state of the participants involved in an ET complex significantly influences the interaction probability.^17,18,24,26^ Thus, in addition to electrostatic contributions from charged residues at the binding interface, having a reduced electron donor and an oxidized electron acceptor helps to initiate formation of the ET complex.^35^ To determine the influence of redox states on the low and high force components of the mutant complexes, SMFS was conducted on oxidized RC-LH1 complexes with both reduced and oxidized cyt *c*_2_ probes at the lowest salt concentration (10 mM NaCl). For the WT and mutant RC-LH1 complexes the interaction probability for the low-force component clearly decreased for the oxidized/oxidized complex compared to oxidized/reduced one (Figure 4A–D). We also calculated the cumulative interaction probabilities from the sum of all interaction probabilities across the range of forces and found a consistent decrease when both partners were oxidized. For the WT RC-LH1 sample, this attenuation was from 34% with reduced cyt *c*_2_ to 32% for the oxidized partner (Figure 4A). The attenuation between the oxidized/reduced and oxidized/oxidized interaction was from 40 to 37% for the N(M188)D mutant (Figure 4B), from 39 to 36% for the Q(L264)E mutant (Figure 4C), and from 24 to 20% for the D(M184)K mutant (Figure 4D). Therefore, the redox state of the participants clearly affects the probability of forming the initial encounter complex.

The low and high force components for the WT (Figure 4A) were shifted toward higher values in the case of oxidized RC-LH1 and oxidized cyt *c*_2_, from 154 (red curve) to 173 pN (orange curve) and 332 pN (green) to 370 pN (olive curve), respectively. At the same time, the intensity of the low-force peak slightly decreased, whereas the intensity of the high-force peak increased. For the mutants, less pronounced changes in the peak positions were observed when interacting with oxidized cyt *c*_2_. However, in this case, there was a noticeable decrease in the intensity of the low-force peaks.

### Brownian Dynamics Simulations of Interactions of Cyt *c*_2_ with the RC-LH1 Complex

To augment the observations from SMFS, we performed three replicas of 500 ns-long BD simulations of the crystallized RC-cyt *c*_2_ complex (PDB: 1L9B), including D(M184)K, Q(L264)E and N(M188)D mutants, and calculated the interaction energies. Thermalized models determined from these simulations revealed that the complex becomes 5- and 20-fold stronger for the N(M188)D and Q(L264)E mutants, respectively, while the interface stability decreases by 3.5-fold in the D(M184)K complex (Figure S5). These computational data are in general agreement with the trends seen in the SMFS experiments (Figures 3 and 4).

**Figure 5:**
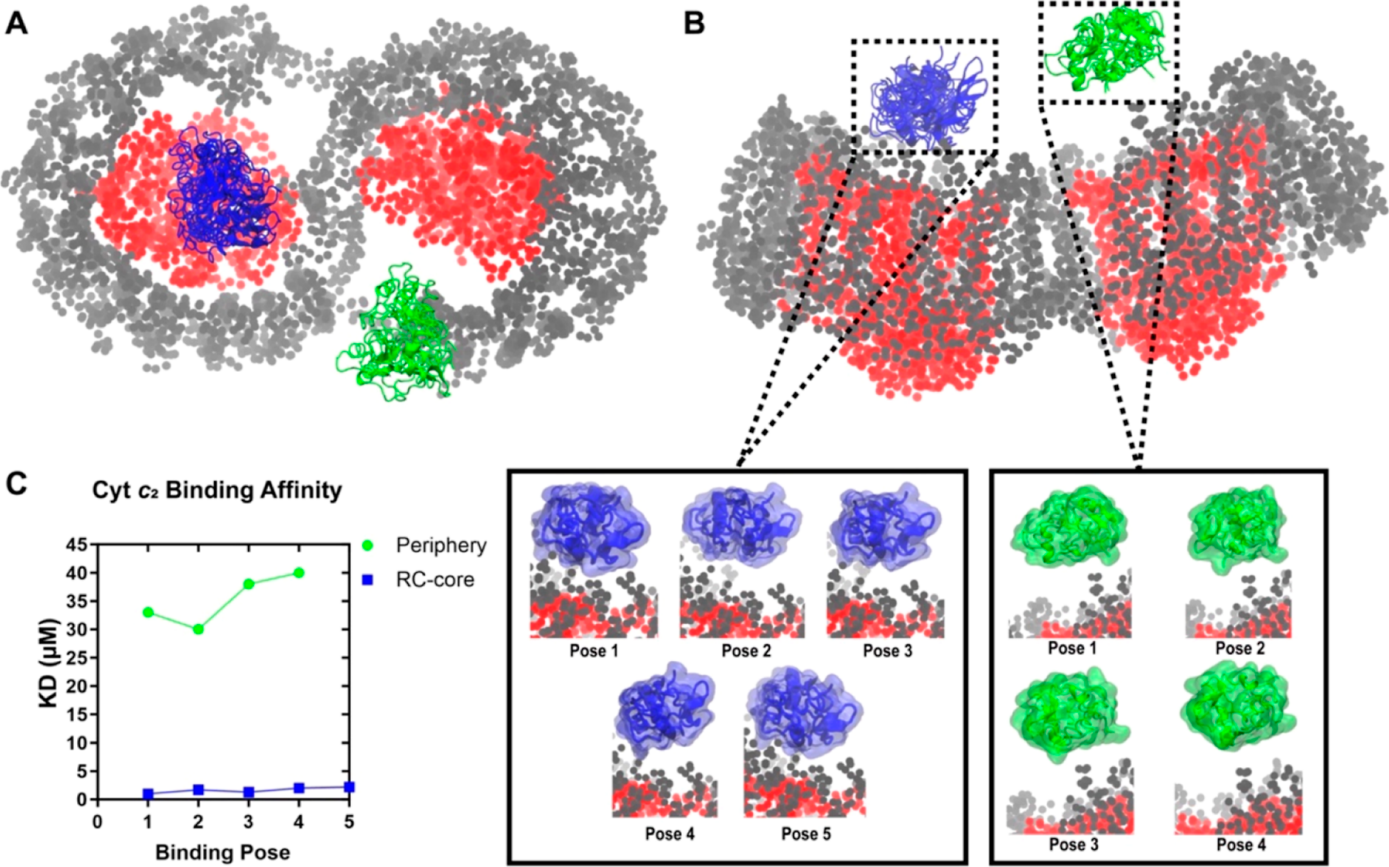
BD simulations of cyt *c*_2_ binding poses on the lumenal surface of the dimeric RC-LH1 complex. (A,B) BD simulation snapshots of a top-down (A) and side view (B) of the proximal (blue) and distal (green) cyt *c*_2_ interaction with the RC core (red) and LH1 periphery (gray). Panel (B) includes a zoomed-in image of the RC/cyt *c*_2_ interaction viewed from the side. (C) The distribution of the calculated *K*_D_ values for each binding pose on the proximal and distal sites.

We then computationally investigated the bimodal RC-LH1/cyt *c*_2_ interactions seen in the SMFS experiments. BD simulations were conducted, as in Singharoy et al.,^22^ and by setting the RC-LH1 complex as oxidized and cyt *c*_2_ as reduced. All binding poses in the cluster were less than 10 Å from the RC or LH1 surfaces. Simulations were repeated for 500 replicas, each of 10 μs, and showed that cyt *c*_2_ interacts with two major clusters on the RC surface, with one site “proximal” to the bacteriochlorophyll special pair and positioned for ET, and another “distal” to the site of ET, closer to the LH1 ring (Figure 5). The Prodigy server^36,37^ was employed to determine the dissociation constants of five proximal and three distal RC-LH1/cyt *c*_2_ complex models, each with the shortest interface distance. *K*_D_ values ranged from 1 to 2.2 μM for the proximally bound cyt *c*_2_. In sharp contrast, the *K*_D_ for the distally bound cyt *c*_2_ is an order of magnitude weaker, in the 30–40 μM range.

### SMFS and Computational Studies of Interactions of Cyt *c*_2_ with RC-Only Complexes

**Figure 6:**
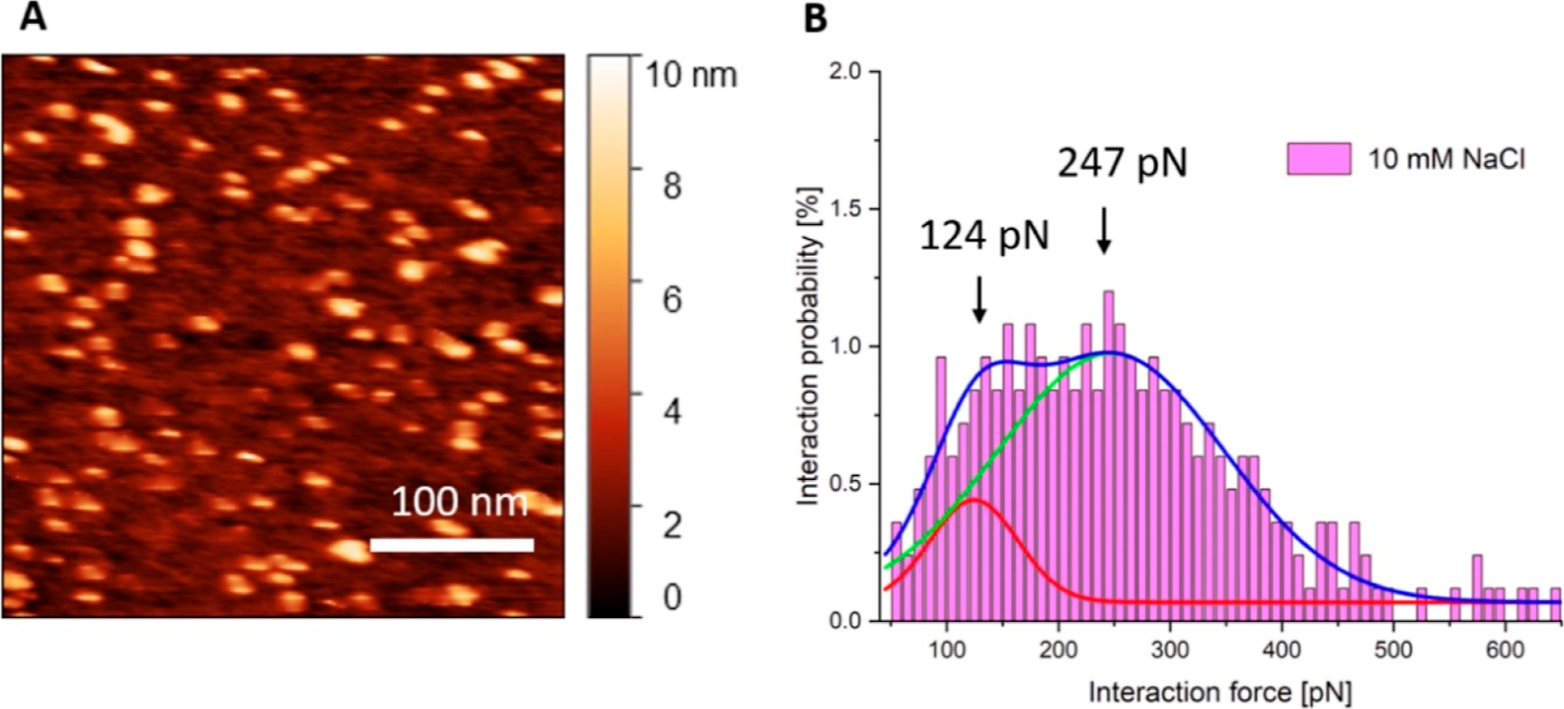
Surface distribution and SMFS interaction force distribution of oxidized RC-only complexes with reduced cyt *c*_2_. Surface topography of immobilized RC complexes (A) and SMFS results (B) showing distribution of the interaction force between the oxidized RC and reduced cyt *c*_2_ complexes versus the interaction probability. The solid curves in (B) represent the best fit for each of the histogram peaks, with the low force component in red, the high force component in green, and the cumulative fit in dark blue. The respective forces, in pN, are shown above each component.

To exclude the possibility that the low force component arises from the LH1/cyt *c*_2_ interaction observed in our BD study, we conducted SMFS experiments that dock cyt *c*_2_ onto the RC complex in the absence of LH1. All SMFS experiments were conducted in a 10 mM NaCl imaging buffer with oxidized RCs and reduced cyt *c*_2_; Figure 6A shows a typical surface distribution of RCs. The SMFS results (Figure 6B) show a similarly bimodal distribution of forces, when compared to the RC-LH1 results (Figures 2–4). Deconvolution gives values of 124 and 247 pN, compared with 154 and 332 pN for the RC-LH1 complex. To investigate the basis for the bimodal SMFS results, we revisited the explicit solvent SMD simulations of the RC - cyt *c*_2_ interaction^13^ in which the RC is in a neutral state and cyt *c*_2_ is in either an oxidized or reduced state. In our new simulations, cyt *c*_2_ is pulled horizontally along the M-subunit and L-subunit of the RC with a velocity of 0.05 Å/ns, (100 times slower than before for improved sampling) for 800 ns.

**Figure 7:**
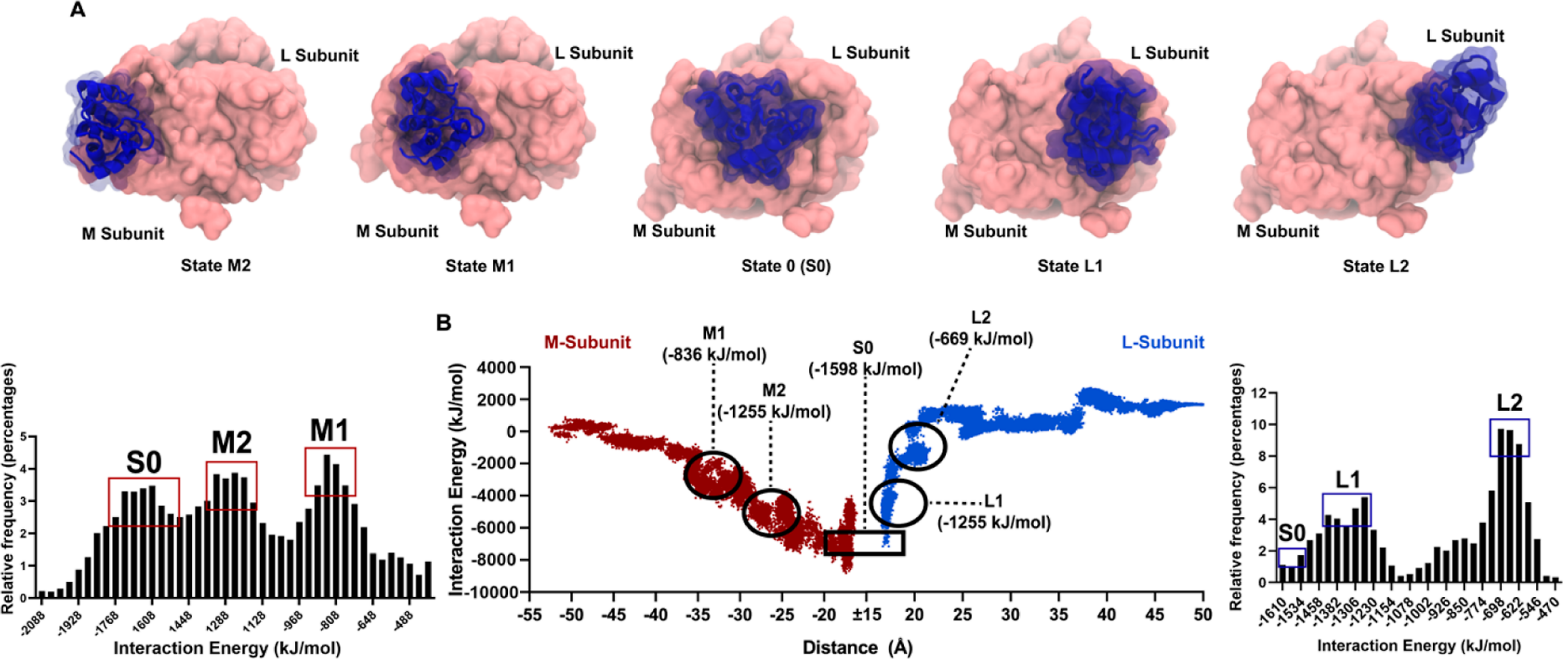
RC-only SMD simulations of cyt *c*_2_ entering along the M-subunit and exiting along the L-subunit. (A) SMD simulation snapshots of a top-down view of cyt *c*_2_ (blue) being pulled horizontally across both the M and L-subunit of the RC (pink). Snapshots correspond with the peak interaction energies on the outsets in (B). Cyt *c*_2_ is in its reduced state when pulling along the M-subunit and oxidized when pulling along the L-subunit. The RC is kept in a neutral state. (B) Distribution of interaction energies between cyt *c*_2_ and RC as the cyt *c*_2_ travels along the entrance pathway (M-side, maroon color) and exit pathway (L-side, blue color). The *x*-axis represents the distance between donor and acceptor. There are two separate energy graphs, one for the entry pathway and the other for the exit pathway, with a central gap corresponding to the S0 state. The two histograms on the outsets show the distribution of interaction energies between (left) reduced cyt *c*_2_ and the RC for the M-side entry pathway, and (right) oxidized cyt *c*_2_ and the RC for the L-side exit pathway.

SMD elucidates changes in interaction energies between cyt *c*_2_ and the RC (Figure 7). Cyt *c*_2_, initially in a reduced state, is pulled along the lumenal surface of the oxidized RC. Figure 7A shows a series of five snapshots from the SMD simulations with cyt *c*_2_ initially on the periphery of the RC distal to the central ET site (state M2), then in an intermediate position (state 1) before arriving at the central ET position (state 0). The red part of the graph in Figure 7B provides a continuous readout of the interaction energies, which increase as cyt *c*_2_ moves along the M-side entry pathway. We see evidence for intermediate states in this energy graph, which are also manifested as peaks in relative interaction frequency (Figure 7B, lower left). The two states along the M-subunit have interaction energies of −1255 kJ/mol (state M2) and −836 kJ/mol (state M1) and correspond to electrostatic interactions between previously identified charged residues K99 on cyt *c*_2_ and D(M184) and E(M95) on the RC,^8,9,13^ with the interaction becoming much stronger as cyt *c*_2_ approaches the site of ET. Thus, the M2 and M1 states identified by our SMD simulations can collectively represent the low force, electrostatic encounter complex revealed by the SMFS experiments. The M-side entrance trajectory culminates in the ET complex, state 0, which is the reference point for merging the M-side and L-side graphs, and the starting point for the L-side exit pathway colored in blue in Figure 7B. As before, we see evidence for intermediate states, and for the exit path we calculated values of −1255 kJ/mol (state L1) and −669 kJ/mol (state L2) for the L-subunit SMD. There are not as many favorable interactions on this exit pathway, apart from a persistent interaction between R32 on cyt *c*_2_ and D(L155) on the RC. Thus, the bimodal force distribution in the SMFS experiments with RC complexes appears to be inherent to the RC and not related to the presence of the LH1 ring. Interestingly, the SMD simulations have uncovered more potential intermediates in the formation pathway than in the dissolution of the RC/cyt *c*_2_ ET complex.

## Discussion

The highly ephemeral nature of the encounter complex has rendered it especially difficult to study via ensemble methodologies.^12^ Here, through continued development of the force spectroscopy approach, which interrogates the interactions between cognate ET partners at the single-molecule level, we have resolved and quantified the forces that establish the encounter and productive ET complexes that form when reduced cyt *c*_2_ docks onto the oxidized RC. This study also builds on previous SMFS measurements of the RC/cyt *c*_2_ ET complex by including the peripheral LH1 antenna, computational simulations, mutations of key evolutionarily conserved residues involved in the electrostatic forces at the binding interface, and variations in the salt concentration of the surrounding medium. Taking the LH1 complex into account brings us closer to the in vivo situation, since RCs are invariably partly or completely surrounded by an arc of LH1 subunits (reviewed in ref (38)).

Previous research has demonstrated the critical role of surface electrostatics in RC/cyt *c*_2_ interactions, most obviously through a series of complementary charges revealed by the RC/cyt *c*_2_ crystal structure,^7,11^ which are investigated further in this study. RC residues D(L155), D(L257), D(L261), E(M95), E(M173) and D(M184) on the lumenal face of the RC can form electrostatic interactions with basic side chains on cyt *c*_2_, which are mainly lysines. The distances involved range from 4.8 to 9.0 Å,^7,11^ but it is also likely that side chains at the lumenal face of the surrounding LH1 ring, comprising 14 αβ subunits in the core RC-LH1 monomer, exert some influence on the approach of cyt *c*_2_ to its docking site on the RC. Although this ring of up to 75 charged side chains (30 Glu, 45 Arg/Lys) is up to 50 Å from the central cyt *c*_2_ binding site, it might nevertheless affect the path of cyt *c*_2_ on its approach to or exit from the RC. Indeed, the BD simulations (Figure 5) predict that cyt *c*_2_ forms a weak “distal” RC-LH1–cyt *c*_2_ complex, with cyt *c*_2_ positioned on the lumenal surface of the 14th LH1 αβ subunit where, in the dimeric complex, the LH1 ring opens to create a gap for quinol export.^29,30^ Given that our SMFS experiments on the RC-LH1/cyt *c*_2_ interaction resolved both low and high force components, it was possible that the lower force component represented the LH1/cyt *c*_2_ distal site identified in the BD simulations. We were able to exclude this by SMFS investigation of the interaction between the naked RC (without LH1) and cyt *c*_2_ (Figure 6) which showed the two force components are inherent features of the RC/cyt *c*_2_ interaction alone, as expected from the RC/cyt *c*_2_ crystal structure.^7^ Most likely, the high (30–40 μM) *K*_D_ for this distally bound cyt *c*_2_ is a sufficiently weak association to elude detection by our SMFS measurements. Nevertheless, this distal binding mode could be functionally significant because confining even a few cyt *c*_2_ molecules within the lumen of a 50 nm diameter chromatophore vesicle is equivalent to a concentration well in excess of 100 mM.^21^ Thus, even this weak, distal LH1 binding site becomes relevant to the overall cycle of cyt *c*_2_-mediated ET, and cyt *c*_2_ could associate peripherally with the LH1 complex as it finally exits the RC-LH1 complex.

The complementary charges come into play as the cyt *c*_2_ moves toward the central region of the RC lumenal face as depicted in Figure 1A. Previous mutagenesis studies and electrostatic calculations show the importance of these conserved residues.^8,10,28,32,33,39,40^ Another important factor is the complementarity between the negative charge on the incoming cyt *c*_2_ heme and the positive charge on the photo-oxidized RC special pair. Previous SMFS studies found that complementary redox states, namely reduced cyt *c*_2_ and oxidized RC, strongly favored formation of an ET complex, whereas this was not the case when both cyt *c*_2_ and the RC were reduced.^17,18^ These findings were consistent with an earlier study showing redox-dependent binding of cyt *c*_2_ to the RC.^35^ Here, we find that the noncomplementary combination of oxidized cyt *c*_2_ and oxidized RC selectively decreases the probability of forming the low force component (Figure 4). Hinting at evolutionary conservation, the importance of complementary redox states for forming the RC/cyt *c*_2_ complex shows clear parallels with the binding of oxidized *Pc* to the reduced cyt *b*_6_*f* complex, where the interaction frequency increased over 5-fold when *Pc* and cyt *b*_6_*f* are in opposite redox states.^24^ Another SMFS study measured the binding of reduced *Pc* to its PSI electron acceptor and showed a higher interaction frequency when at least one of the partners is in a redox state ready for ET, along with lower binding for the post-ET (reduced PSI and oxidized *Pc*) pair.^24,26^ Thus, redox gating is clearly a general feature which exists to avoid unproductive encounters between ET partners and thereby maintain the rapid turnover of ET chains throughout the domains of life.

Interestingly, the low force component identified in our study is also more strongly attenuated by increasing salt concentration (Figure 2B–D), and more affected by alteration of key charged residues on the RC relative to the high force component. We found that the probability of forming the low force component is decreased in the D(M184)K RC mutant and increased in the Q(L264)E and N(M188)D complexes (Figure 3), consistent with the respectively decreased and increased number of potential electrostatic interactions with the incoming cyt *c*_2_. Considering the acute effects of salt and redox state on the low force component we suggest that it reflects formation of the “encounter complex”, which is promoted and steered by electrostatic forces, which can be considered as long-range, with a boundary of >20 Å.^10^ The SMD simulations add more granularity, resolving the encounter complex into two components, M2 and M1, snapshots of which are shown in Figure 7A. These various states now warrant further investigation via site-directed mutagenesis and SMFS.

Following encounter complex formation, reorganization into a productive RC-LH1/cyt *c*_2_ ET complex must occur. This requires closer contact between reactants to facilitate efficient ET, with a RC BChl special pair to cyt *c*_2_ heme distance of 8.4 Å.^7^ The BD simulations (Figure 5C) show that the *K*_D_ associated with this tighter binding of cyt *c*_2_ is in the 1–2 μM range, consistent with studies of ET kinetics that determined a value of 0.3 μM.^8,28^ Here, we note that our SMFS experiments dock reduced cyt *c*_2_ onto the surface of the oxidized RC with a tip-sample contact time of 70–240 μs, which is comparable with the half-life time of the bound state of the ET complex.^34^ Our SMFS measurements show that a relatively high force is required to separate the productive ET complex, resolved as a ∼330 pN component (Figures 2-4). This higher force component was clearly less affected by increasing salt concentration consistent with an additional contribution of short-range hydrophobic interactions. Indeed, the formation of the productive ET complex, state 0 in Figure 7, is characterized by a series of nonpolar interactions with cyt *c*_2_ on the M-subunit side of the lumenal face of the RC.^7,13^ The RC/cyt *c*_2_ structure shows that an important contact for ET was between a heme methyl group and the ring of Y(L162) of the RC, and showed the functional importance of this tyrosine residue.^7,13,41^ Many other nonpolar contacts were identified in the vicinity of Y(L162), as well as van der Waals and cation–π interactions.^7^ In addition, several water molecules were resolved at the RC/cyt *c*_2_ docking interface, the long-lived behavior of which was examined in earlier MD simulations.^9,13^ These interface water molecules appear to act differently for the oxidized and reduced redox states of cyt *c*_2_. Thus, we conclude that our SMFS experiments also partly reflect the behavior of functionally important interface water molecules. MD simulations, and the earlier crystallographic study of the RC/cyt *c*_2_ complex,^7,13^ showed that the salt bridges formed between oppositely charged RC and cyt *c*_2_ residues are partially solvated ensuring that no tight contacts are made, which favors the eventual dissociation of cyt *c*_2_. Indeed, past simulations show that the post-ET ingress of water molecules could weaken the interface between the reduced RC and oxidized cyt *c*_2_ by disrupting electrostatic contacts.^13^ Our SMD simulations (Figure 7) show that there are L1 and L2 intermediates in the L-side exit path taken by oxidized cyt *c*_2_ after it leaves the S0 ET site. Undocking in this direction allows its replacement by an incoming reduced cyt *c*_2_ destined to form an initial encounter complex above RC-M.^13^ We note that this undocking pathway would involve oxidized cyt *c*_2_ moving toward the distal site identified in our BD simulations (Figure 5). Our single molecule results also chime with recent work by Gomila et al.,^42^ who studied the interaction between mitochondrial cyt *c* and complex III. In this work, introduction of a single negative charge to the cyt *c* binding interface via tyrosine phosphorylation was sufficient to increase the unbinding force and slow ET between the partners by slowing overall turnover.

In summary, our results show that it is possible to resolve and quantify the encounter and productive ET complex states of the RC-LH1/cyt *c*_2_ interaction using SMFS. This approach can therefore facilitate a more in depth understanding of the importance of the encounter complex in the future and is now ripe for translation to other photosynthetic and respiratory ET systems.

## Materials and Methods

### Protein Purification

The strains of *Rba. sphaeroides* used in this work, Δ*puc1BA puhA*-His_10_ (U1) and Δ*puc1BA* Δ*pufBALMX puhA*-His_10_ (T9), were made by adding the sequence encoding a His_10_-tag to the 3′ end of the *puhA* gene, which encodes the reaction center H subunit, in the strains Δ*puc1BA* and Δ*puc1BA* Δ*pufBALMX*. A fragment homologous to 406 bp prior to the stop codon of *puhA* was ligated into pET-52b(+) (Novagen) with XbaI and NotI. Subsequently, this fragment and the in-frame, plasmid-encoded thrombin linker and His_10_-tag sequence, was amplified and ligated into pK18mob*sacB* along with a fragment homologous to 395 bp downstream of the stop codon of *puhA*. The resulting plasmid was introduced to the target strains by conjugative mating from *Escherichia coli* strain S17-1. The homologous regions surrounding the His-tag allowed a two-step recombination to insert the tagged sequence in the target position.^43,44^ The correct tagging of the *puhA* gene was confirmed by PCR and automated Sanger sequencing (Eurofins).

The point mutation in *pufM* encoding D184 K was introduced by creating a pK18mob*sacB* plasmid by overlap-extension PCR of fragments containing ∼400 bp upstream and downstream of the target residue, with the desired mutation introduced at the overlap region. This was conjugated into strain U1 and screened by PCR and sequencing to identify colonies containing the mutation. The other mutations, encoding PufM N188D and PufL Q264E, were provided on pRK*::pufBALMX* plasmids by Professor Michael R. Jones (University of Bristol, UK). These plasmids were conjugated into the T9 strain using 20 μg/mL tetracycline as a selectable marker.

RC-LH1 complexes were purified as described previously.^17,18^ Briefly, semiaerobically grown cells were harvested and disrupted in a French pressure cell at 18,000 psi. After centrifugation, the supernatant was loaded onto a sucrose gradient in order to isolate the intracytoplasmic membranes (ICM). After harvesting, the ICMs were solubilized in 3% (v/v) β-DDM by stirring in the dark at 4 °C for 45 min. The solubilized membrane solution was diluted at least 3-fold in the working buffer and centrifuged for 1 h (160,000*g*) at 4 °C to remove nonsolubilized material. RC-LH1 complexes were isolated from the supernatant by using immobilized metal ion affinity and size-exclusion chromatography. The purified protein complexes were concentrated to a final concentration in the range of 15–45 μM in 10 mM HEPES pH 7.4, 10 mM NaCl, 0.59 mM β-DDM buffer and stored at −80 °C for further use. His-tagged RC-only complexes were purified by detergent exchanging RC-LH1 complexes into 1% LDAO and washing, followed by anion exchange on DEAE Sepharose in 50 mM Tris pH 8 with 0.1% LDAO. After confirming LH1 removal by spectrophotometry, RCs were buffer exchanged back into 10 mM HEPES pH 7.4, 10 mM NaCl, 0.59 mM β-DDM buffer, concentrated in the range of 15–45 μM and stored at −80 °C for further use.

The cyt *c*_*2*_ gene *cycA* was modified to encode a His_6_ sequence^17,18^ for immobilized metal ion affinity chromatography. The ICM fraction from the His-tagged cyt *c*_*2*_ mutant was fractionated on a sucrose gradient and the membrane pellet was solubilized using *N*,*N*-dimethyldodecan-1-amine oxide (LDAO, Sigma) at a final concentration of 65 mM, and a final OD of the membrane sample of ∼80 at 875 nm at room temperature in the dark for 20 min. After removing the nonsolubilized material by centrifugation, the protein was purified using immobilized metal ion affinity. The purified protein (*A*_414_/*A*_280_ ≥ 3.3) was dialyzed against 10 mM HEPES pH 7.4, 10 mM NaCl, 1 mM LDAO buffer, concentrated to a final concentration of approximately 700 μM and stored at −80 °C for further use.^18^

### Protein Immobilization on the AFM Probes and SiOx Substrates

The functionalization of the AFM probes with cyt *c*_*2*_ and the immobilization of the RC-LH1 complexes were performed employing a method described earlier.^17,18^ Briefly, the SiOx substrates and the AFM probes (BL-AC40TS-C2, Olympus Probes) were cleaned in Piranha solution (H_2_O_2_/H_2_SO_4_ 1:2 (v/v)) for 1 h, rinsed 10 times in deionized water and finally dried in a pure nitrogen stream. Immediately after the cleaning step, both the Si substrates and the AFM probes were placed into a glass desiccator and were coated with self-assembled monolayer (SAM) of 3-mercaptopropyltrimethoxysilane (MPTMS, Sigma-Aldrich) by using simple vapor-phase deposition method, where both the AFM probes and the Si substrates were placed into a vacuum desiccator in the presence of 20 μL of MPTMS and left for 6–8 h to facilitate the deposition of the SAM. The next step in the functionalization of the AFM probes, immediately after the SAM formation, was to introduce a heterobifunctional cross-linker, complemented by a 9.5 nm long polyethylene-glycol (PEG) spacer on the AFM probe, which in turn allowed us to introduce Ni^2+^-NTA functionality (AB-NTA, Dojindo Laboratories) on the surface of the AFM probe and the SiOx substrates to bind the His-tagged protein complexes. The Ni^2+^-NTA coordination chemistry enables strong and durable attachment of proteins to surfaces and AFM probes, allowing the acquisition of several thousand force–distance cycles over the course of several hours.^17,18^ The surface density of the RC-LH1 complexes on the SiOx substrate was approximately 300–500 molecules per 1 μm^2^.

### Affinity Mapping Measurements and Data Analysis

All the experiments described here were performed using a Multimode 8 instrument equipped with a NanoScope V (Bruker) controller. NanoScope (v 9.2) software (Bruker) was used for data collection. All the AFM measurements were performed in imaging buffer (10 mM HEPES pH 7.4) supplemented with NaCl at different concentrations. The measured spring constants of the BL-AC40TS cantilevers were in the range 0.087–0.262 N m^–1^. The *Z*-modulation amplitude was adjusted to values in the range 20–25 nm to allow enough tip-sample separation to fully stretch the PEG linker molecule on the AFM tip and to separate the RC-LH1 and the cyt *c*_*2*_ complexes during each ramp cycle. The contact tip-sample force was kept in the range 60–100 pN and the imaging rate was adjusted (depending on the scan size and pixel density of the scan) to ensure a consistent number of force–distance curves recorded per image pixel. Experimental conditions were maintained for the duration of the force–distance curve acquisition to ensure consistency in our imaging over an extended period. The AFM probes and/or the samples were washed every 5–7 min in reducing imaging buffer (supplemented with 0.5 mM sodium dithionite and 0.25 mM phenazine methosulfate) or in oxidizing buffer. This procedure was followed after acquiring two to three AFM scans, which equates to approximately a few tens of productive interactions between the redox partners on the AFM probe and sample surface. Accordingly, at any given time during our experiments, there was a regularly replenished pool of prereduced cyt *c*_2_ proteins on the AFM probe. For the control experiments, the docking site of the RC-LH1 complexes on the substrate was blocked by injection of a 10-fold molar excess of free, prereduced cyt *c*_*2*_ directly into the AFM imaging cell. Alternatively, the AFM probes functionalized with cyt *c*_*2*_ were chemically preoxidized (treated with 0.8 mM potassium ferricyanide solution) and then washed in the imaging buffer. All the AFM data were analyzed by using Gwyddion v 2.61 (open-source software covered by GNU general public license, www.gwyddion.net), Nanoscope Analysis (Bruker), and OriginPro (OriginLab Corp.) software. Gwyddion and Nanoscope Analysis were used for image processing and analysis. Nanoscope Analysis was also used for the extraction and analysis of the SMFS data. OriginPro was used for the statistical analysis of all the force spectroscopy data and data visualization. For the force data in our manuscript, the instantaneous values of the loading rate measured just prior to the rupture event on the curve are broadly distributed around a most probable loading rate of approximately 2.5 × 10^6^ (±4.67 × 10^5^) pN s^–1^.

### Steady-State Assays of Cytochrome Oxidation

Turnover assays were conducted under steady state conditions in a similar fashion to that described earlier,^38^ using 10 μM reduced cyt *c*_*2*_, 50 μM ubiquinone-2 (Sigma) and 0.5 μM reduced RC-LH1 in a buffer mixture containing 50 mM Tris at pH 7.5, 100 mM NaCl and 0.03% w/v β-DDM. Following overnight dark adaption, each reaction mixture was placed in a quartz cuvette and the oxidation state of cyt *c*_2_ was monitored at 550 nm using a Cary 60 spectrophotometer (Agilent Technologies). After 10 s in the dark, excitation energy was delivered via a fiber optic cable from an 810 nm M810F2 LED (light-emitting diode) (Thorlabs, UK) driven at 100% intensity using a DC2200 controller (Thorlabs Ltd., UK). The data were processed by fitting the linear initial rate over 26 ms, starting from the first data point where the absorbance started dropping continuously.

### Brownian Dynamics Simulations

BD simulations were used to monitor the motions of quinone and cyt *c*_2_ molecules on the time scale of hundreds of microseconds using an in-house-developed GPU-accelerated BD simulation code, atom resolved brownian dynamics.^45^ BD simulations of cyt *c*_2_ were performed over a cumulative time of 30 ms at pH 7 and 250 mM salinity, for both the reduced and oxidized RC. These simulation conditions exemplify the microenvironmental stresses that single-ET proteins overcome while shuttling charges across a bioenergetic membrane. For each condition, 500 independent 10 μs trajectories were generated with a single cyt *c*_2_ molecule starting at the center of the RC. The cyt *c*_2_ molecules were modeled as rigid body particles. At each time step, a torque and force were evaluated based on the configuration of the system, allowing the update of position and orientation for the rigid body using a symplectic integrator. The mass and moments of inertia of cyt *c*_2_ were calculated directly from the atomic coordinates. The program Hydropro was used to estimate the diagonal components of the diffusion tensor from the atomic coordinates by replacing each surface atom with a sphere subject to Stokes drag. The diffusion tensor was then transformed to translational and rotational friction coefficients according to the Stokes–Einstein relation. These provided Langevin forces and torques at each time step that kept the system at 300 K. The Lennard-Jones parameters of the atoms comprising the cyt *c*_2_ were clustered into three categories: one representing all hydrogen atoms, another representing oxygen and nitrogen atoms, and the final representing carbon and sulfur atoms. The atoms in each category were assigned an average value for the parameters *R*_min_ and ε, which were used to calculate a potential for the interaction of such an atom with the entire RC using the implicit ligand sampling feature of VMD at 1 Å resolution. The potentials, both electrostatic and Lennard-Jones, were smoothed by a 1 Å wide 3D Gaussian filter to remove noise. These potentials and the corresponding densities were used to perform five hundred 10 μs simulations with cyt *c*_2_ initially placed at the center of the RC. A 100 fs time step was used and the cyt *c*_2_ coordinates were recorded every nanosecond for subsequent analysis. To obtain transition times and overall affinity, the cyt *c*_2_ and any given RC membrane protein were considered to be in contact after any pair of non-hydrogen atoms from the two proteins came within 7 Å of one another. The contact was considered lost once no pair of atoms from the proteins was found within 16 Å. Contact with the lipid bilayer was similarly defined, except that contacts with the lipid bilayer were not tracked on a per-molecule basis.

### MD Simulations

The RC–cyt *c*_2_ complex for *Rba*. *sphaeroides* (PDB: 1L9B) was set up in a POPC membrane of hexagonal geometry with the TIP3 water model as described in Pogorelov et al.,.^13^ The D(M184)K, Q(L264)E and N(M188)D RC mutants were made using the Mutator plugin of VMD. Equilibration was performed in the *NPT* ensemble with a cutoff of 12 Å for van der Waals interactions and periodic boundary conditions with a flexible cell were applied. A time step of 1 fs was used at a constant temperature of 298 K and constant pressure of 1 atm. Heme parameters were specified using CHARMM27^46^ and MD simulations were performed for 500 ns, with three repeats for each mutation. The system size was 144,709 atoms with the RC–cyt *c*_2_ complex, membrane, water, and ions. Interaction energies were calculated using the NAMD Energy plugin in VMD. For the steered molecular dynamics (SMD) simulations, cyt *c*_2_ was pulled along the M-subunit and L-subunit for ∼50 Å. The forces and spring constant are the same as described in Pogorelov et al.,.^13^ Cyt *c*_2_ was pulled along the M-subunit and L-subunit with a velocity of 0.05 Å/ns, which is 100 times slower than in Pogorelov et al.,^13^ to allow for improved sampling of intermediate states. Time evolution of the force and displacement of the center of mass of cyt *c*_2_ was recorded every 0.1 ps. Visualization of trajectories and calculation of interaction energies were performed using VMD and its NAMD Energy plugin. Simulations were carried out using ASU’s Sol Supercomputer and Oak Ridge Leadership Computing Facility’s Frontier Supercomputer.

## Data Availability

The accession number for the modeling, simulation and analysis input files used in this article is MendeleyData: https://data.mendeley.com/preview/bckptcyz5f?a=639b6855-d824-4c94-b151-8e3efe55a74b.

## Supporting Information Available

Supplementary distribution of interaction force histograms, dependence of cytochrome *c*2 oxidation rate on NaCl concentration and interaction energies calculated by molecular dynamics (PDF)
