# Supplementary material for: Single-Molecule Detection of the Encounter and Productive Electron Transfer Complexes of a Photosynthetic Reaction Center
Source: J Am Chem Soc. 2024 Jul 11;146(29):20019–32. doi: 10.1021/jacs.4c03913 (PMC11273609; doi:10.1021/jacs.4c03913)
Supplement: Supplementary file 1 — ja4c03913_si_001.pdf [file ja4c03913_si_001.pdf]

Single-molecule detection of the encounter and productive electron transfer complexes of a photosynthetic reaction centre

Cvetelin Vasilev[1], Jon Nguyen[2], Adam G.M. Bowie[1], Guy E. Mayneord[1], Elizabeth C. Martin[1], Andrew Hitchcock[1], Taras V. Pogorelov[3], Abhishek Singharoy[2*], C. Neil Hunter[1*] and Matthew P. Johnson[1*]

[1]Plants, Photosynthesis and Soil, School of Biosciences, University of Sheffield, Firth Court, Western Bank, Sheffield, S10 2TN, UK
[2]School of Molecular Sciences, Arizona State University, Tempe, 85281, USA
[3]Department of Chemistry, Center for Biophysics and Quantitative Biology, Beckman Institute for Advanced Science and Technology, National Center for Supercomputing Applications, School of Chemical Sciences, University of Illinois Urbana-Champaign, Urbana, IL 61801, USA

*= authors for correspondence matt.johnson@sheffield.ac.uk; c.n.hunter@sheffield.ac.uk; asinghar@asu.edu

Supporting Information for publication

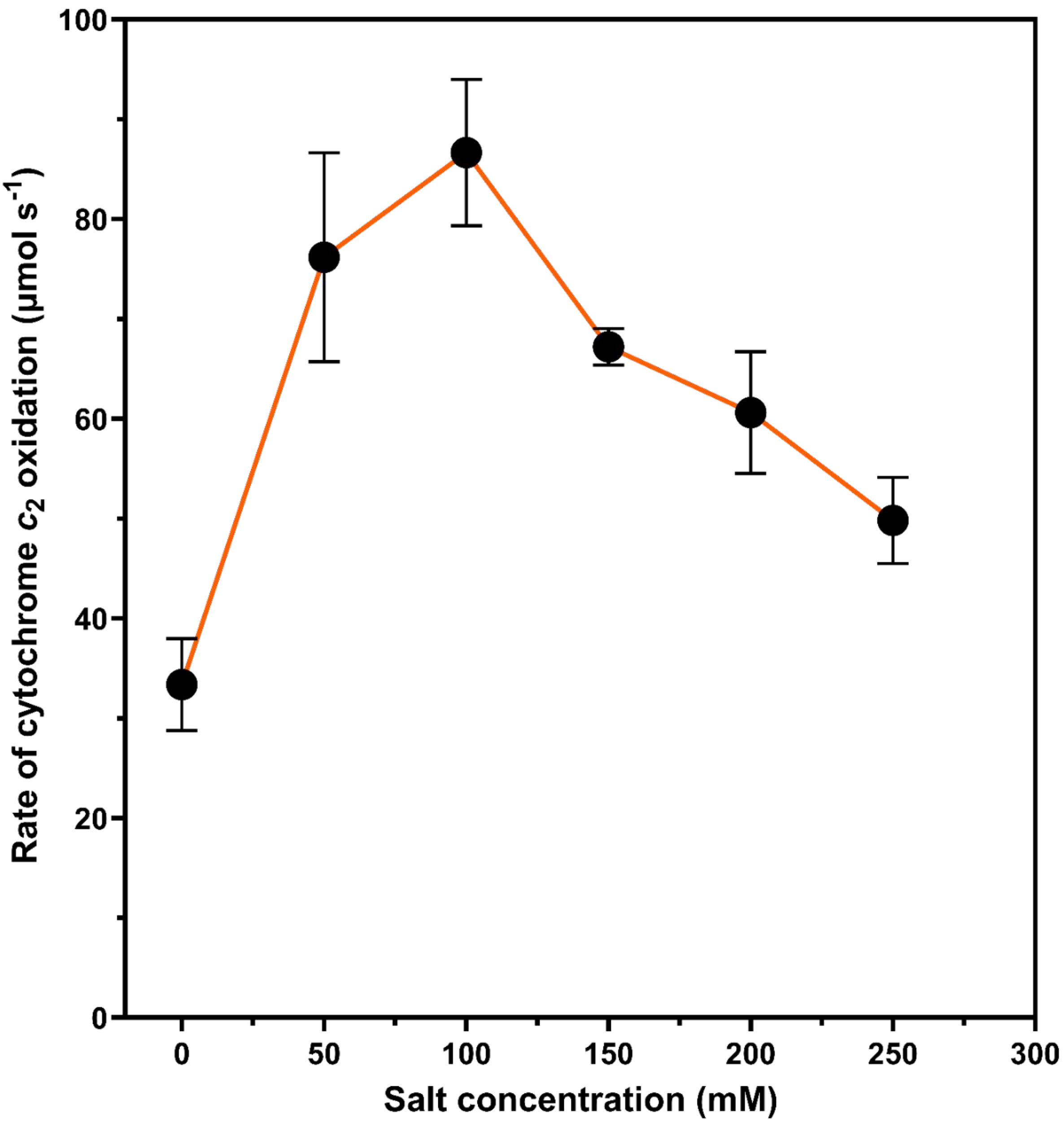

Figure S1. Effect of NaCl concentration on the steady state rate of cytochrome c_2 oxidation by the RC-LH1 complex. This rate was calculated from the change in absorbance at 550 nm for the first 25 milliseconds of the reaction following illumination. Whilst the optimum salt concentration appears to be approximately 100 mM, these data suggest the RC-LH1 complex remains highly active over a broad range of salinities, even up to 200 mM and likely beyond.

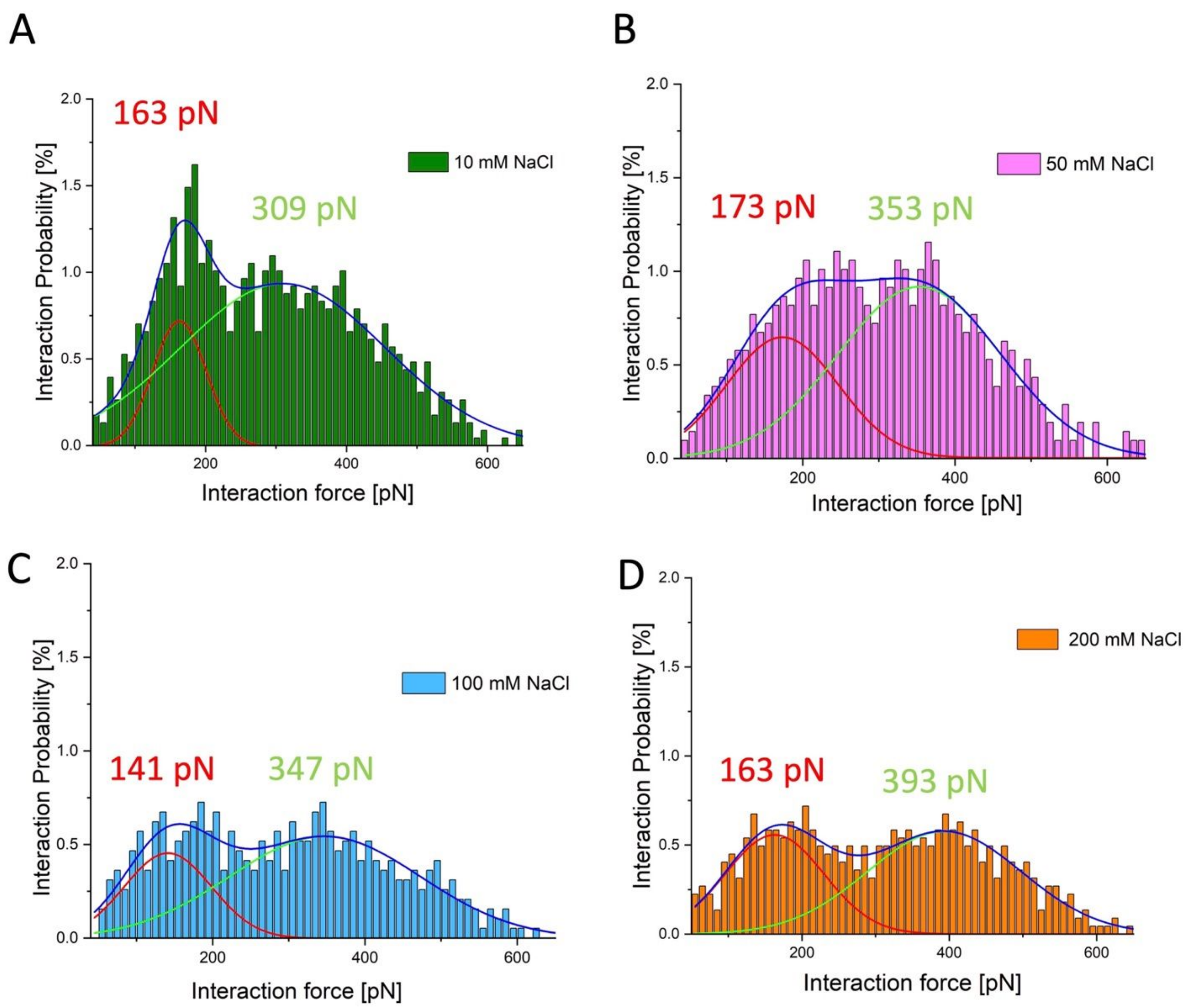

Figure S2. Distribution of the interaction force between oxidized Q(L264)E RC-LH1 complexes and reduced cyt c_2 versus the interaction probability. The histograms represent the distribution of the interaction forces measured by SMFS as oxidized Q(L264)E RC-LH1 complexes and reduced cyt c_2 are brought into contact and then separated. **(A-D)** The salt concentration in the imaging buffer was increased from 10 mM to 200 mM, as indicated next to each histogram. The solid curves represent the best fit for each of the histogram peaks, with the low force component in red, the high force component in green, and the cumulative fit in dark blue. The respective forces, in pN, are shown above each component.

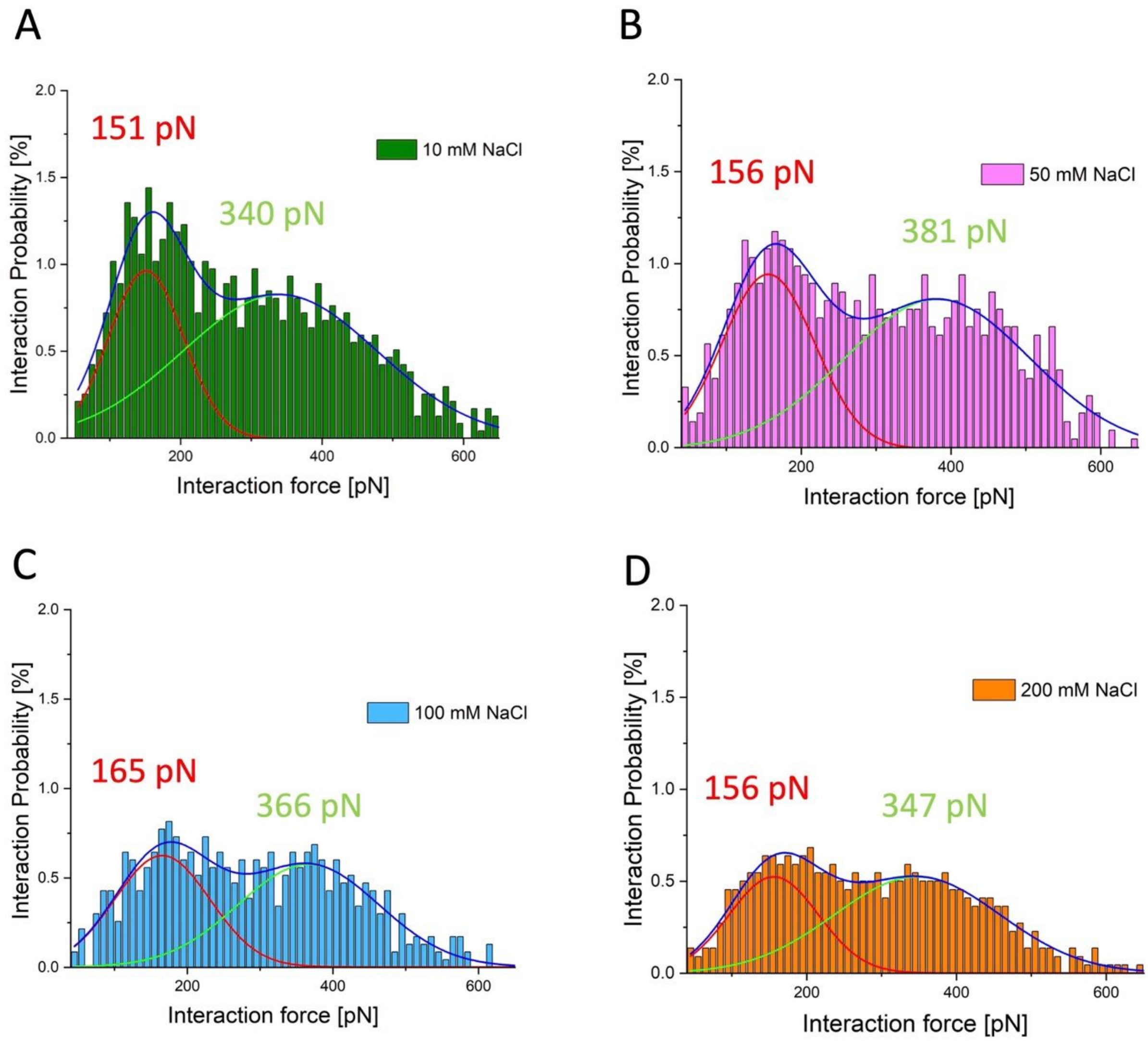

Figure S3. Distribution of the interaction force between oxidized N(M188)D RC-LH1 complexes and reduced cyt c_2 versus the interaction probability. The histograms represent the distribution of the interaction forces measured by SMFS as oxidized N(M188)D RC-LH1 complexes and reduced cyt c_2 are brought into contact and then separated. **(A-D)** The salt concentration in the imaging buffer was increased from 10 mM to 200 mM, as indicated next to each histogram. The solid curves represent the best fit for each of the histogram peaks, with the low force component in red, the high force component in green, and the cumulative fit in dark blue. The respective forces, in pN, are shown above each component.

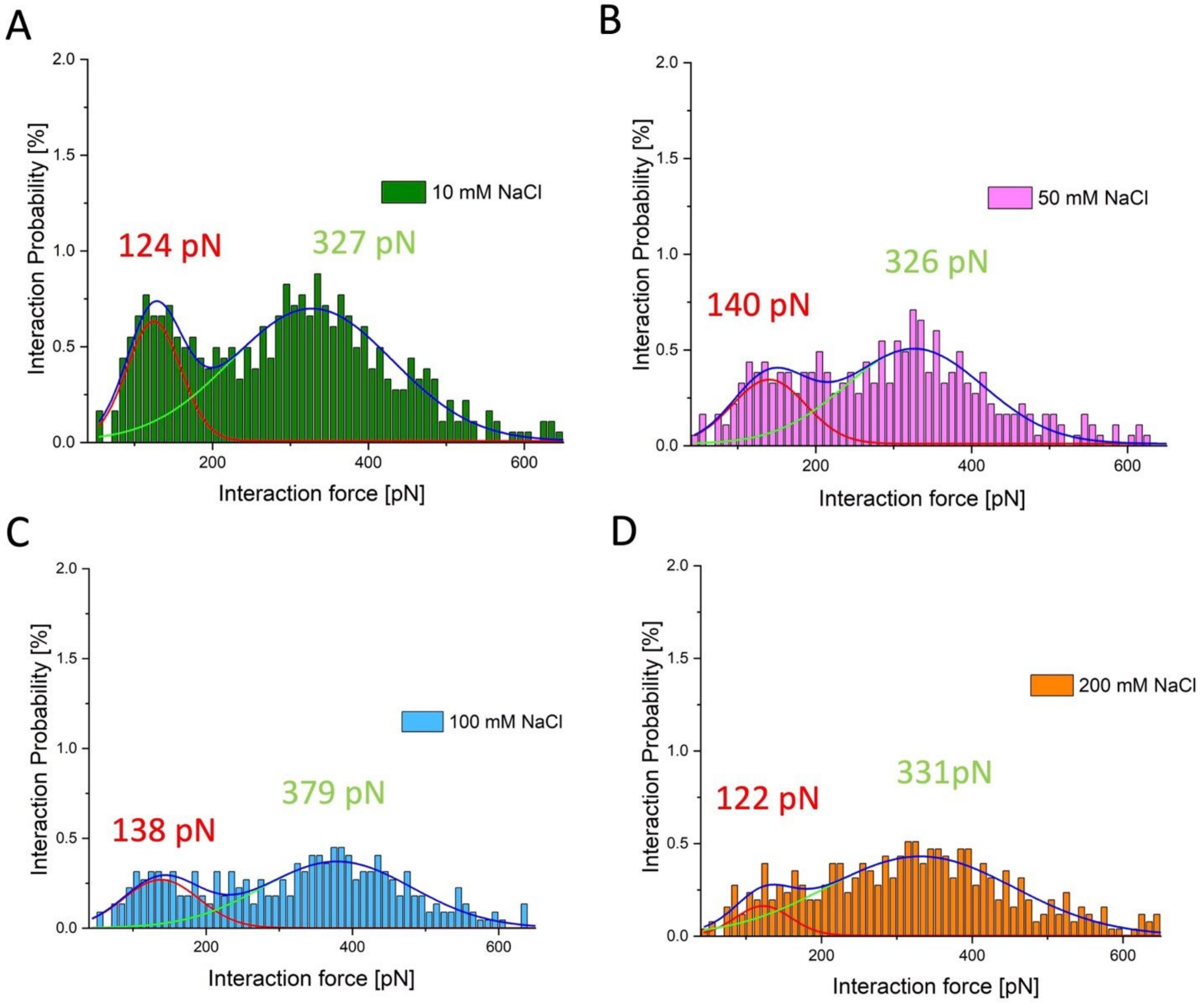

Figure S4. Distribution of the interaction force between oxidized D(M184)K RC-LH1 complexes and reduced cyt c_2 versus the interaction probability. The histograms represent the distribution of the interaction forces measured by SMFS as oxidized D(M184)K RC-LH1 complexes and reduced cyt c_2 are brought into contact and then separated. **(A-D)** The salt concentration in the imaging buffer was increased from 10 mM to 200 mM, as indicated next to each histogram. The solid curves represent the best fit for each of the histogram peaks, with the low force component in red, the high force component in green, and the cumulative fit in dark blue. The respective forces, in pN, are shown above each component.

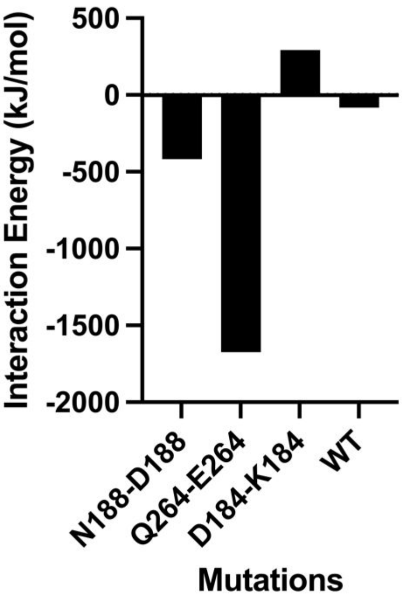

Figure S5. Interaction energies for WT and mutant RC-cyt c_2 complexes calculated from molecular dynamics simulations.
